# FetA Antibodies Induced by an Outer Membrane Vesicle Vaccine Derived from a Serogroup B Meningococcal Isolate with Constitutive FetA Expression

**DOI:** 10.1371/journal.pone.0140345

**Published:** 2015-10-14

**Authors:** Holly Sanders, Gunnstein Norheim, Hannah Chan, Christina Dold, Caroline Vipond, Jeremy P. Derrick, Andrew J. Pollard, Martin C. J. Maiden, Ian M. Feavers

**Affiliations:** 1 National Institute of Biological Standards and Control, South Mimms, Potters Bar, United Kingdom; 2 Oxford Vaccine Group, Department of Paediatrics, University of Oxford, and the NIHR Oxford Biomedical Research Centre, Oxford, United Kingdom; 3 Division of Infectious Disease Control, Norwegian Institute of Public Health, Oslo, Norway Faculty of Medicine, University of Oslo, Oslo, Norway; 4 Faculty of Life Sciences, The University of Manchester, Michael Smith Building, Oxford Road, Manchester, United Kingdom; 5 Department of Zoology, University of Oxford, Oxford, United Kingdom; Universidad Nacional de la Plata, ARGENTINA

## Abstract

Invasive meningococcal disease causes over 3500 cases each year in Europe, with particularly high incidence among young children. Among serogroup B meningococci, which cause most of the cases, high diversity in the outer membrane proteins (OMPs) is observed in endemic situations; however, comprehensive molecular epidemiological data are available for the diversity and distribution of the OMPs PorA and FetA and these can be used to rationally design a vaccine with high coverage of the case isolates. The aim of this study was to determine whether outer membrane vesicles (OMVs) derived from an isolate with constitutive FetA expression (MenPF-1 vaccine) could be used to induce antibodies against both the PorA and FetA antigens. The immunogenicity of various dose levels and number of doses was evaluated in mice and rabbits, and IgG antibody responses tested against OMVs and recombinant PorA and FetA proteins. A panel of four isogenic mutants was generated and used to evaluate the relative ability of the vaccine to induce serum bactericidal activity (SBA) against FetA and PorA. Sera from mice were tested in SBA against the four target strains. Results demonstrated that the MenPF-1 OMVs were immunogenic against PorA and FetA in both animal models. Furthermore, the murine antibodies induced were bactericidal against isogenic mutant strains, suggesting that antibodies to both PorA and FetA were functional. The data presented indicate that the MenPF-1 vaccine is a suitable formulation for presenting PorA and FetA OMPs in order to induce bactericidal antibodies, and that proceeding to a Phase I clinical trial with this vaccine candidate is justified.

## Introduction

Invasive meningococcal disease is a severe and life-threatening acute bacterial infection, with highest incidence among children less than 5 years of age [1]. The majority of invasive meningococcal disease in Europe, the Americas and Australasia is caused by *Neisseria meningitidis* expressing the serogroup B capsule (MenB) [1], for which no polysaccharide-based vaccine is available. The sialic acid residues in the MenB capsule are structurally similar to human neural-cell adhesion molecules, and both poor immunogenicity and concern over generating autoimmunity prevent further development of a MenB polysaccharide vaccine [2,3]. Vaccine prevention of MenB disease has focused on the subcapsular antigens as vaccine candidates.

The first-generation vaccines designed to prevent MenB disease are based on detergent extracted outer membrane vesicles (OMVs) from wildtype epidemic strains and able to prevent disease caused by homologous strains in all age groups [4]. Such vaccines have been used against lineage-specific epidemics of MenB disease [5,6,7,8,9], but mainly provide protection against the lineage responsible for the epidemic through antibodies directed against the variant of the immunodominant PorA antigen present [10].

To provide broader protection, effective against multiple meningococcal lineages, multiple variants and preferably several antigens have been targeted. Major challenges are that immunogenic epitopes on these antigens are usually highly variable among meningococcal strains, whilst more conserved antigens are either transiently expressed, poorly immunogenic, or are incapable of inducing bacterial clearance when tested in *in vitro* assays [11]. Vaccine development strategies have included using (i) single variants of multiple antigens, for example the 4CMenB vaccine (Bexsero^®^, Novartis, Sienna, Italy) [12]; or (ii) many variants of a single antigen, for example the proposed NonaMen vaccine (National Institute for Public Health and the Environment, Bilthoven, The Netherlands) [13]. There are advantages and disadvantages to each approach. Using a single antigen may limit coverage as protection is unlikely to be induced against all variants equally [14], and minimal protection will be induced against non-vaccine variants. With cocktails of multiple antigens, antibodies to less immunogenic or less abundant antigens may act synergistically to result in bacterial clearance [15,16]; however, PorA is the immunodominant protein in the meningococcal outer membrane and in comparison the immune response evoked by other antigens is often inferior. Other proteins which have shown good immunogenicity may not be expressed at high levels by all strains for example NadA [17].

PorA and FetA (alternatively called FrpB) are two major outer membrane proteins. FetA is an iron transporter [18]. The crystal structure of FetA shows that the region of antigenic variation is concentrated into a sub-domain which protrudes above the predicted location of the outer membrane, and hence is accessible to antibody. PorA is a porin protein, likely to have a similar structure to PorB [19] where hypervariable regions extend from beta strands in the 16-stranded barrel. Both PorA and FetA are known to be immunogenic: antibodies to both proteins have been found in convalescent sera [20,21], suggesting that these antigens are also expressed in vivo.

Rational design of a vaccine that covers the majority of MenB disease isolates requires extensive epidemiological data for the chosen vaccine components. Such data are already available for PorA and FetA [22]. The study of the distribution of PorA and FetA variants within meningococcal populations show them to be structured with hyperinvasive lineages associated with particular variants of each antigen [23] which are stable for prolonged periods of time [24]. It was estimated that a combination of variants of PorA and FetA could provide high levels of coverage against invasive meningococcal disease in Europe [22,25]. The population structuring fits a model driven and maintained by competition between meningococcal lineages for hosts and the development of immunity by those hosts [23]. The model suggests that antibodies to both proteins are involved in natural protection against meningococcal carriage and disease.

Consequently, the knowledge of the structuring of meningococcal populations can be used to rationally design a multivalent vaccine. OMVs, have previously been shown to effectively induce protective antibodies against PorA. However, generating a reliable antibody response against FetA expressed in OMVs from wildtype strains is potentially more difficult, as FetA expression is highly inconsistent *in vitro* due to negative regulation by iron [26,27].

We have developed an OMV vaccine derived from a MenB strain genetically-modified to constitutively express FetA. Here we investigated whether the MenPF-1 vaccine could induce serum bactericidal activity (SBA) against both the PorA and FetA proteins simultaneously in a proof-of-concept mouse model. For this, a panel of meningococcal isogenic mutants was generated for use as SBA target strains, allowing a delineation of the specificity of the SBA responses. Immunogenicity in rabbits, following a toxicology study, is also presented as a prerequisite to regulatory approval for a Phase I study.

## Materials and Methods

### Bacterial strains

Bacterial strains used are described in Table 1.

**Table 1:** Meningococcal strains used in this study.

Strain	Genotype	PorA expression	FetA expression	Source
H44/76	*porA*, *fetA* Wild-type	Wild-type	Wild-type	Norwegian Institute for Public Health
SMenPF1.2	H44/76 *fetAp*_*17bp*_	Wild-type	Constitutive	This study
3043	H44/76 *fetA*::*kan*	Wild-type	Absent	[28]
3311	H44/76 *fetA*::*kan porA*::*ermC*	Absent	Absent	This study
3312	H44/76 *fetAp*_*17bp*_ *porA*::*ermC*	Absent	Constitutive	This study

Expression levels of major outer membrane proteins PorA and FetA are indicated.

**Fig 1:**
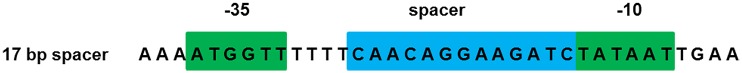
Sequence of the modified *porA* promoter region used to replace the native *fetA* promoter. Bases constituting the -10 and -35 regions are highlighted in green. Bases taken from the *porB* promoter are highlighted in blue.

**Fig 2:**
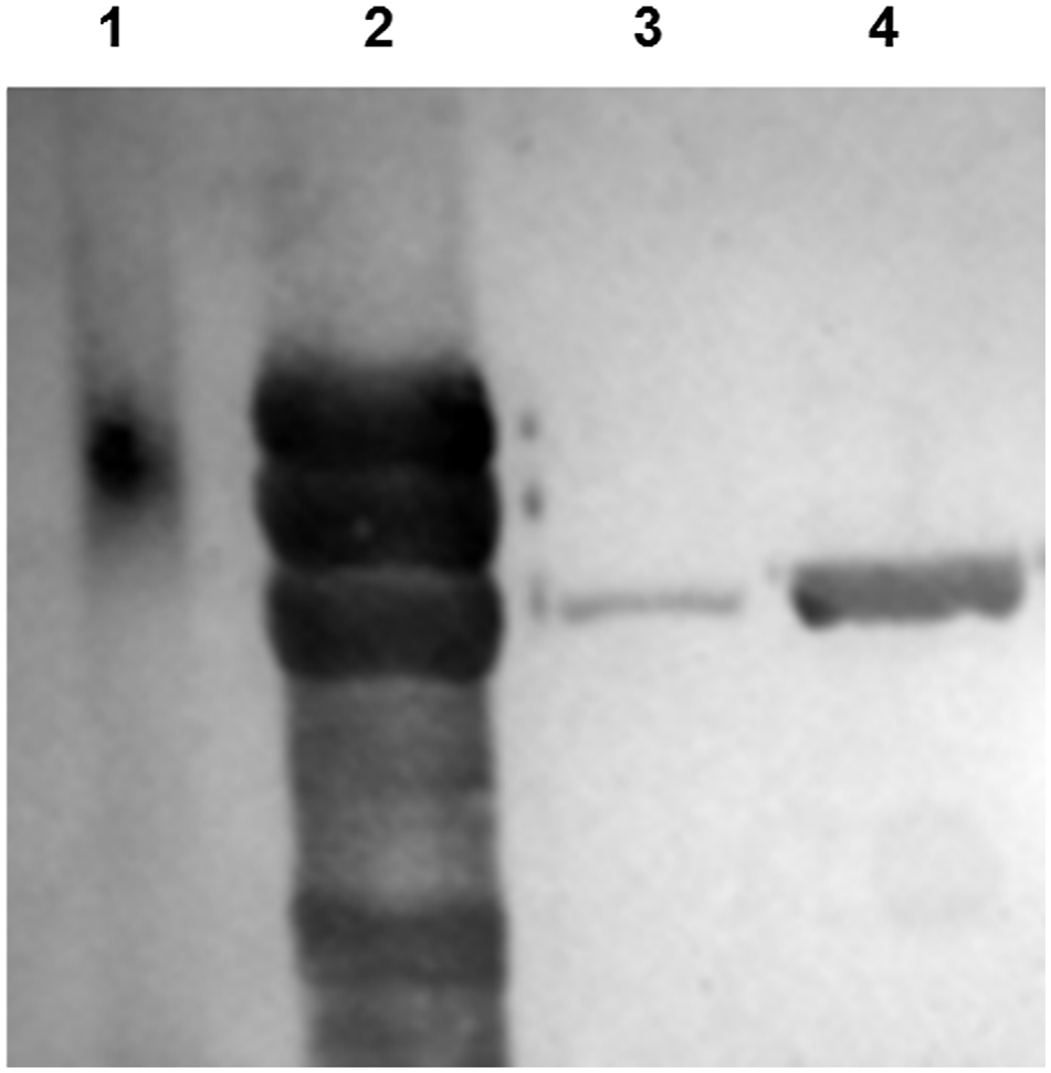
Verification of FetA expression in wildtype (H44/76) and genetically modified strain (SMenPF1.2). Analysis by immunoblot (antiserum raised to recombinant FetA variant F3-3) of SDS-PAGE gel with 10 μg protein (outer membrane protein preparations) per well. BioRad pre-stained SDS broad-range standards (70kDa standard shown in Lane 1). Lane 2: Purified FetA F3-3. Lane 3: Wildtype H44/76. Lane 4: Constitutive Strain (SMenPF1.2).

The SMenPF1.2 strain is a modified version of the MenB H44/76 strain (B:15:P1.7,16) with wild-type expression of PorA and constitutively high expression of FetA as a result of replacement of the native promoter by transformation. SMenPF1.2, was genetically modified whereby the promoter upstream of the *fetA* gene was replaced with a modified promoter derived from sequences upstream of the *porA* and *porB* genes (Fig 1), to increase FetA expression and remove its regulation by iron. Increased expression of FetA in SMenPF1.2 compared to the wildtype strain was confirmed by Western blot using a FetA polyclonal serum raised to recombinant FetA (Fig 2).

Strain 3043 had previously been generated by transformation of strain H44/76 with a plasmid pEAT2 which contains the FetA gene interrupted at an *Eco*O109I site by a kanamycin resistance cassette to interrupt expression of the FetA protein (S1 Fig) [28].

The genetically-modified *N*. *meningitidis* strains SMenPF1.2 and 3043 were used both as SBA target strains and as parent strains in order to generate two additional isogenic mutants for use as SBA target strains. To generate the two new mutants (3311 and 3312) with interrupted expression of the full-length PorA protein, strains SMenPF1.2 and 3043 were transformed with plasmid PorAEryF (provided by Ojas Mehta, CCVTM, Oxford, UK), S2 Fig. This plasmid contains the *porA* gene interrupted at a *Bsi*WI restriction site with an erythromycin-resistance cassette sub-cloned from plasmid pER2 [29]. Meningococci were re-suspended from overnight growth on blood agar (Oxoid, Cambridge, UK) to an OD 650nm of 0.2–0.3 in 1 mL Mueller Hinton broth (Oxoid) containing 8 mM MgCl_2_ (Sigma Aldrich, St Louis, Missouri, USA). Undigested plasmid DNA (1μg) was added and the suspension was incubated at 37°C with 150 rpm rotational shaking for 4 hours. Cells were collected by centrifugation and plated on to Mueller Hinton agar (Oxoid) containing 50 μg/mL erythromycin (Sigma Aldrich) to select for plasmid integration. To verify the functional effect of the genetic modification, whole cells of the strain panel were tested in dot-blot with a PorA-specific monoclonal antibody (Anti-P1.16 01/538, NIBSC, Potters Bar, UK) or a FetA-specific mouse antiserum (generated by immunization of mice with recombinant FetA).

### Meningococcal OMV vaccine (MenPF-1)

**Fig 3:**
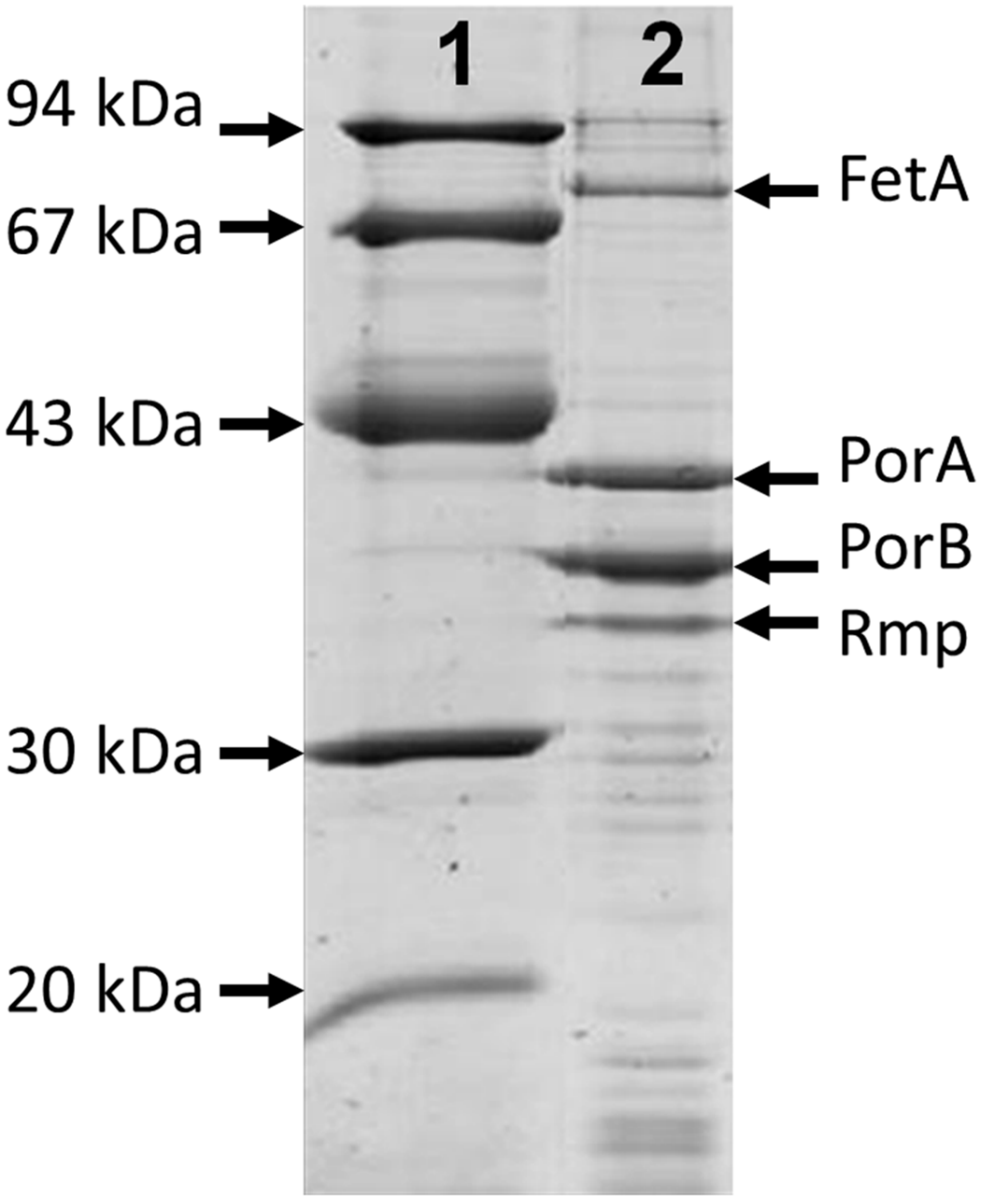
Characterisation by SDS-PAGE of the MenPF1 outer membrane vesicles used for immunization. Lanes: 1) Molecular weight protein markers. 2) OMVs from strain SMenPF1.2.

OMVs were produced from strain SMenPF1.2 by the Norwegian Institute of Public Health (Oslo, Norway) by extraction with deoxycholate [30]. The OMVs (active ingredient) contained 21.8% PorA protein, 7.7% FetA protein (as determined by scanning densitometry relative to total protein content) and 0.03 μg LPS per μg protein (3%, as determined by silver staining SDS-PAGE gels, using a quantitative L3,7 standard) (Fig 3). The vaccine product MenPF-1 is a sterile filtered OMV solution, formulated with Al(OH)_3_ (1:66 ratio of protein:Al(OH)_3_ (w/w)), sucrose (25 mg/mL) and water for injection (WFI) in sealed, capped and labeled injection vials (0.6 mL per vial including 0.1 mL overfill). A final dose vial contained 50 μg OMV protein per mL.

SDS-PAGE of OMV samples was performed as described previously [31].

### Immunization of mice

Animal studies were conducted according to the UK Home Office regulations under licence number 80/2157 and were approved by the National Institute for Biological Standards and Control ethics committee. Final adsorbed MenPF-1 vaccine (≥0.5 mL per vial) was diluted to the required concentration (2, 5, 10 or 20 μg protein/mL) in 145 mM sodium chloride solution (Sigma Aldrich) immediately before use. Groups of 10 (adjuvant-only control) or 20 female NIH/OlaHsd mice (18–22 g, Harlan Laboratories, Blackthorn, UK) were immunized subcutaneously with 500 μl of diluted vaccine suspension at 3 week intervals. For a dose response study, mice were given two doses of 1.0 μg, 2.5 μg, 5.0 μg, or 10.0 μg of total protein per dose. A concomitant study of the number of doses was conducted whereby mice were given 1, 2, 3 or 4 doses of 2.5 μg OMVs. Bleeds were collected by cardiac puncture under terminal anaesthesia three weeks after the last dose. Blood was allowed to clot at 4°C for 18 hours. Serum was separated by centrifugation at 9600 x g for 10 minutes and stored at -20°C until use. Sera from 20 mice given 2 doses of 10 μg total protein per dose were pooled for use as a standard serum in immunological assays.

To provide sufficient volume of sera for comparison against all target strains in SBA, sera from groups of four mice were pooled resulting in five pools per treatment group.

### Immunization of rabbits

Rabbit sera used in this study was obtained from a repeated-dose toxicity study of the MenPF-1 final vaccine (OMVs adsorbed to aluminium hydroxide), performed by Charles River Laboratories (Edinburgh, UK). Briefly, New Zealand White rabbits (12 weeks at start of dosing, Harlan, UK) were given four doses of vaccine or control over a 9 week period (Days 1, 22, 43 and 64). Rabbits were given either an adjuvant-only control, a single human dose (25 μg of OMV with Al(OH)_3_ adjuvant), or a double human dose (2 x 25 μg of OMV with Al(OH)_3_ adjuvant). For each immunization, six male and six female animals were used; six animals in each group were euthanized on day 66 (main study group), while the remaining six animals were assigned to a “recovery group” and euthanized on day 92 to assess for delayed toxicity. Blood samples for immunological analysis were taken pretrial (sample 1) and before dosing on days 22 (sample 2), 64 (sample 3) and 92 (sample 4; recovery group). Serum was separated by centrifugation at 1500 x g for 10 minutes and stored at -20°C until use.

In addition, a polyclonal rabbit antiserum pool was generated as a reference for assay standardization. Four female New Zealand White rabbits (14 weeks old, Harlan, UK) were immunized subcutaneously with 25 μg of OMV with Al(OH)_3_ adjuvant on days 0 and 21. Blood was collected on day 42. Sera from all four rabbits were pooled to generate the standard serum.

### Enzyme-linked immunosorbent assay (ELISA)

To quantify antibodies in animal sera against OMVs, bulk OMVs were diluted to 2 μg/mL in 15 mM sodium carbonate / 35 mM sodium bicarbonate (both Sigma Aldrich) buffer, pH 9.6. 100μl per well was used to coat 96-well flat-bottomed microtitre plates (NUNC, Thermo Fisher, Massachusetts, USA) overnight at 4°C. Plates were washed with PBS (137 mM Sodium chloride/2.7 mM potassium chloride/10 mM disodium hydrogen phosphate/1.8 mM potassium dihydrogen phosphate/pH adjusted to 7.4 with hydrochloric acid, all Sigma Aldrich) containing 0.01% (v/v) Tween-20 (Polyethylene glycol sorbitan monolaurate, Sigma Aldrich). All further dilutions were made in PBS/5% fetal calf serum (Life Technologies, Paisley, UK). All incubations were for 60 minutes at room temperature, followed by washing of the plates. Plates were blocked with dilution buffer, and serial dilutions (1:3) of sera or positive control were added. After incubation, anti-goat anti-mouse or anti-rabbit IgG horseradish peroxidase conjugate (Sigma Aldrich) was added at a 1:2000 dilution. To detect the secondary antibody, plates were incubated for 5 minutes with 100 μl per well tetramethylbenzidine ELISA substrate (Universal Biologicals, Cambridge, UK). 100 μl per well 1M sulphuric acid (Sigma Aldrich) was added to stop the reaction. Absorbance at 450 nm was recorded using a Multiskan MS plate reader (Labsystems, Helsinki, Finland). IgG concentrations (in arbitrary units), compared to the standard serum pool (either mouse or rabbit), were calculated using CDC ELISA software (CDC, Atlanta, USA). The standard serum pool was given a value of 10 arbitrary units.

Recombinant PorA and FetA antigens for ELISA were provided by Hema Patel and refolded from inclusion bodies using protocols described elsewhere [32,33]. ELISAs against recombinant proteins were completed as above, with the following modifications: for coating of plates, proteins were diluted to 0.5 μg/mL in PBS; for dilution of sera and secondary antibodies PBS/0.5% (w/v) bovine serum albumin/0.01% (v/v) Tween-20 was used.

### Serum bactericidal assays (SBA)

SBA were performed as described previously [34] with the exception of using Gey’s balanced salt solution (rabbit complement SBAs) or Hanks balanced salt solution with magnesium and calcium (HBSS, for human complement SBAs,) with 0.5% (w/v) BSA (both Sigma Aldrich) as the bactericidal buffer. Sera were tested from a final dilution (after the addition of meningococci and complement) of 1:4 (human complement) or 1:8 (rabbit complement) with two-fold serial dilutions to 1:1024 or 1:2048, respectively, with higher dilutions tested where necessary. Baby rabbit serum was used as an exogenous source of complement (Pel-Freeze Biologicals, Arkansas, USA). For human complement SBAs, two human donors were identified as suitable sources. Sera from donor 345, donations 2 and 3 were used for meningococcal strains 3312 and 3043 respectively. Sera from donor 352, donations 2 and 3 were used for meningococcal strain SMenPF1.2 and 3311 respectively. All complement sources were used at a final concentration of 25%. The SBA titre assigned was the reciprocal of the serum dilution that resulted in 50% killing compared to the viable count (or the midpoint between two dilutions if 50% killing was determined to be between the two dilutions). Sera were assigned a titre of 2 where 50% killing was not achieved at a dilution of 1:4.

### Statistical analysis

Statistical analysis of ELISA data was performed using SPSS software (Version 20, International Business Machines Corp, New York, USA). Minitab 17 (Minitab. Inc, State College, PA, USA) was used for the analysis of SBA log transformed titres by fitting general linear models with meningococcal strains, dose levels or number of doses as factors, with post-hoc Tukey’s or Dunnett’s tests (using SMenPF1.2 as a control group to compare against 3043 and H44/76) used where appropriate.

## Results

### Generation of meningococcal isogenic mutants for vaccine evaluation

**Fig 4:**
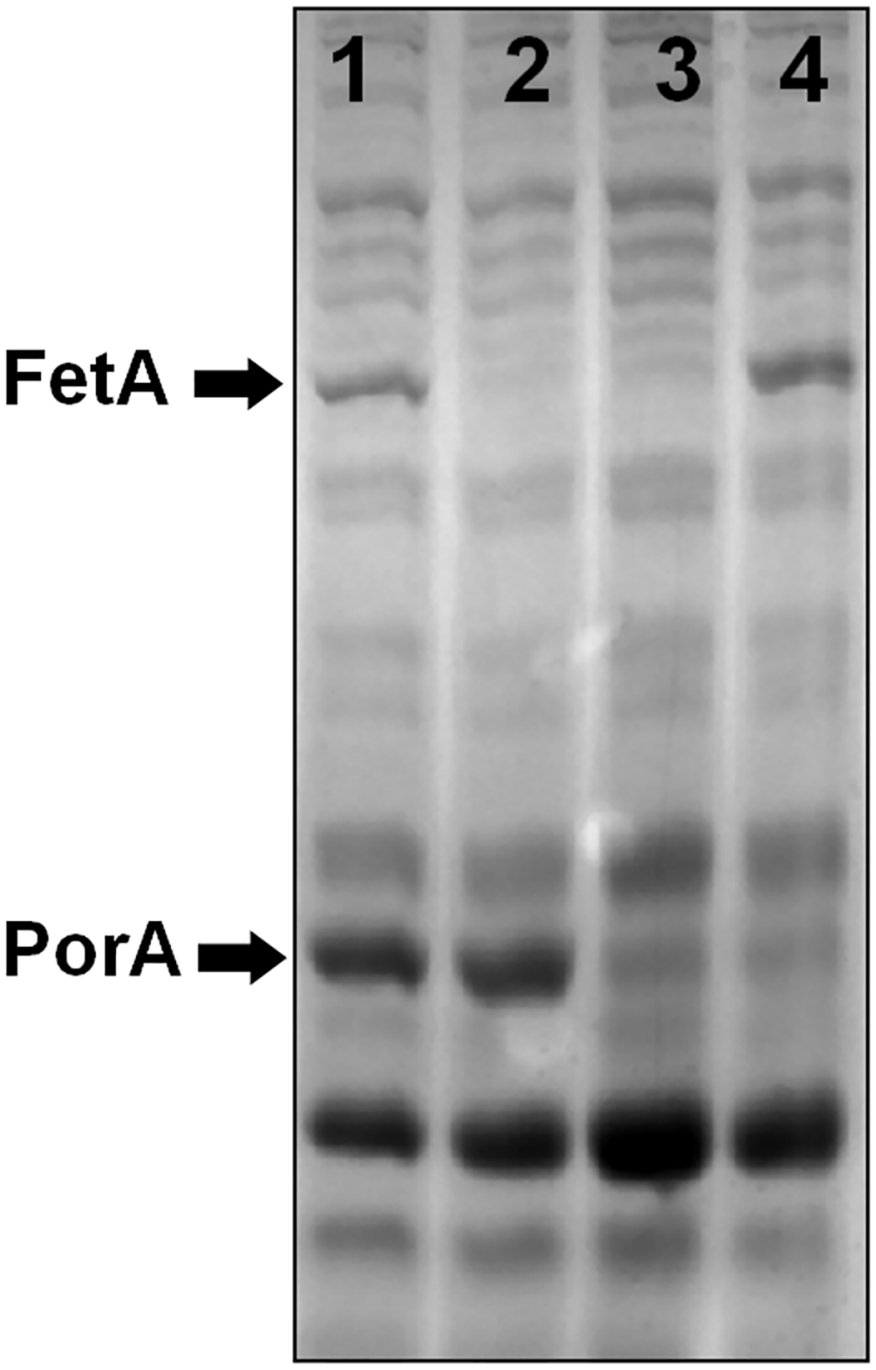
SDS-PAGE of outer membrane proteins from the isogenic mutant strains used to evaluate the serum bactericidal response. 50 μg total protein was loaded per lane. Lanes: 1) SMenPF1.2 (*fetAp*_*17bp*_), 2) 3043 (*fetA*::*kan*), 3) 3311 (*fetA*::*kan porA*::*ermC*), 4) 3312 (*fetAp*_*17bp*_ *porA*::*ermC*).

As the aim of this study was to determine whether OMVs produced from the modified strain could be used to induce antibodies against both the PorA and FetA antigens, a total of four strains was developed in which expression of the two antigens was present or absent, in various combinations (Table 1). The OMV production strain, strain SMenPF1.2, expressed PorA at wild-type levels and FetA constitutively at increased levels compared to the repressed wildtype. An isogenic H44/76 mutant, strain 3043, in which expression of FetA had been interrupted was also available [28]. Strain SMenPF1.2 was further modified by interruption of the *porA* gene to produce strain 3312, in which FetA was expressed constitutively in the absence of PorA expression. Similarly, the *porA* gene was interrupted in strain 3043, resulting in strain 3311, in which expression of both FetA and PorA were absent (Table 1). These strains showed minimal differences in growth and expression of other proteins compared to wildtype H44/76 (Fig 4). Expression of PorA and FetA was confirmed in the vaccine strain by dot blot with specific PorA anti-serosubtype P1.16 monoclonal antibody and a polyclonal antibody raised against FetA variant F3-3 (data not shown).

### Immunogenicity of OMVs in mice

**Fig 5:**
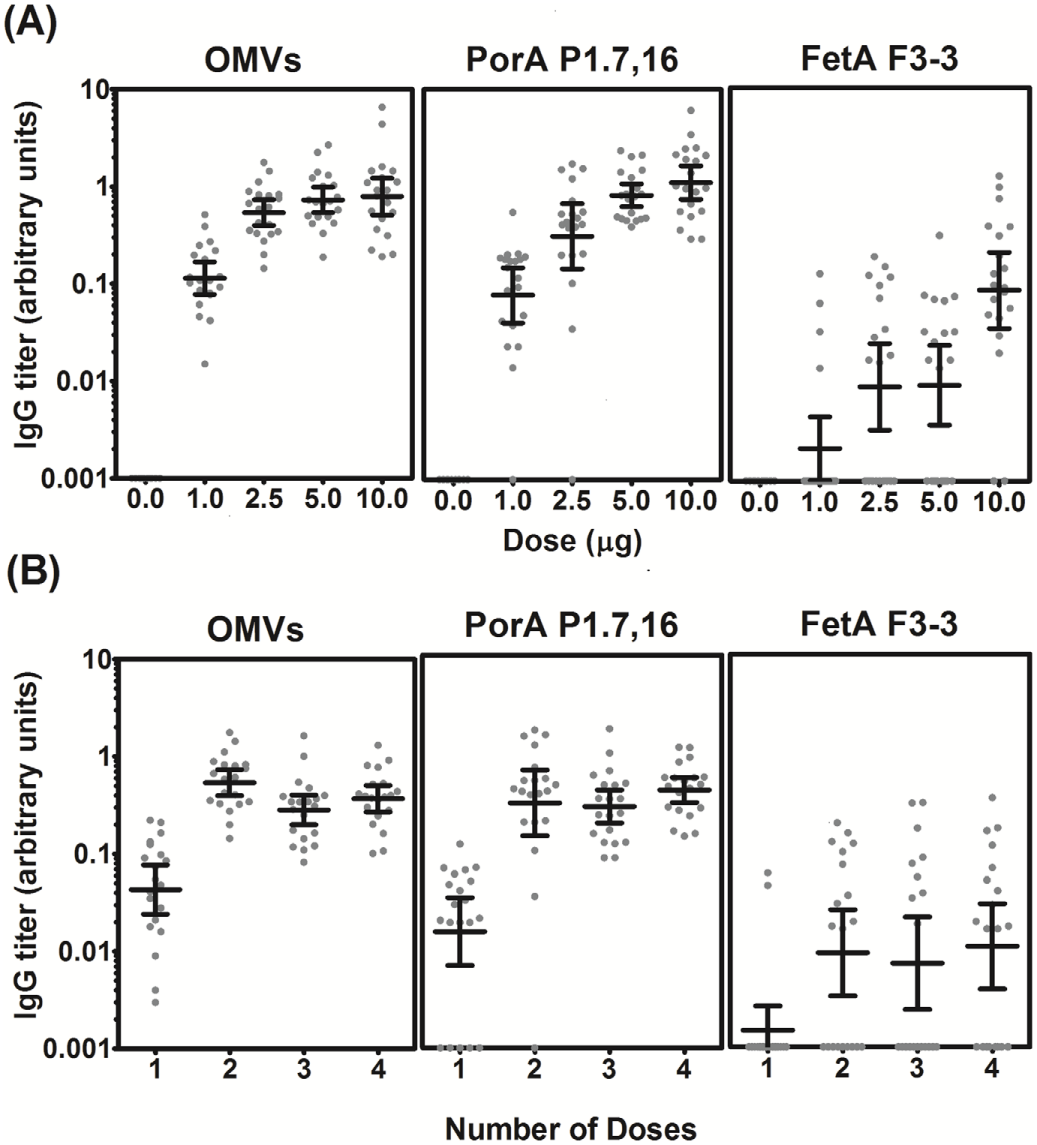
Immunogenicity of MenPF-1 vaccine in mice. Total IgG titres, calculated in comparison to a pooled standard serum, were determined for sera from individual mice against bulk OMVs and the PorA and FetA antigens. The standard serum was given an arbitrary value of 10 units. The geometric mean IgG titres are indicated with a line. Error bars show the 95% confidence intervals of the mean. (A) Mice given two doses of four dose levels or an μg protein per doses. Note that the data from mice given two doses of 2.5 μg protein per dose is included in both figures for comparison, as the immunizations were performed concomitantly. Actual figures for the data presented are given in S1 Table.

The immunogenicity of the SMenPF1.2 OMVs was investigated in mice using multiple dose levels. Mice were given two doses of 1 μg, 2.5 μg, 5 μg, or 10 μg of total protein per dose. ELISA analysis showed an increase in levels of total IgG against the bulk OMVs, PorA and FetA (Fig 5A). Dose responses to both PorA and FetA were observed, although the responses to FetA were low: for all antigens the concentration of IgG measured in sera from mice given 10 μg protein per dose was significantly higher than from mice given 1 μg protein per dose (Bulk OMV P < 0.001; PorA P < 0.001; FetA P < 0.001). No antibodies were observed in sera from mice given an adjuvant-only control.

The functional activity of the antibodies was analyzed using SBA assays performed against panel of four isogenic target strains. SBAs or titres determined using rabbit or human complement sources are referred to in the text as rSBA or hSBA, respectively. Interaction between strain and either dose levels or number of doses was not significant for all statistical analyses.

High levels of bactericidal activity using rabbit or human complement, against the strain used to produce the OMV vaccine were demonstrated at all dose levels tested (Tables 2 and 3). Similar bactericidal antibody titres were measured against strain 3043 (*fetA*::*kan*) as the target, but titres to both these strains expressing PorA were significantly higher against the *porA*::*ermC* strains 3311 and 3312. Using a human source of complement in the SBA, GMTs against the modified vaccine strain SMenPF1.2 were significantly higher than those obtained against the wildtype, unmodified strain H44/76, P < 0.001 (Table 3).

**Table 2:** Immunogenicity (rSBA) of MenPF-1 vaccine in mice (dose levels 1.0–10.0 μg per dose).

Dose	Serum Bactericidal Titre (GMT) rabbit complement			
μg, (2 doses)	SMenPF1.2 *fetAp*_*17bp*_	3043 *fetA*::*kan*	3312 *fetAp*_*17bp*,_ *porA*::*ermC*	3311 *fetA*::*kan*, *porA*::*ermC*
1.0	**1383 (921–2078)**	**1233** (817–1862)	**10** (5–19)	**5** (4–6)
2.5	**2097 (1415–3107)**	**795** (341–1854)	**25** (14–43)	**7** (5–10)
5.0	**2767 (1982–3862)**	**3566** (2942–4322)	**12** (10–14)	**11** (6–17)
10.0	**1933** (1197–3123)	**1723** (1252–2372)	**16** (12–22)	**13** (9–19)

Serum bactericidal titres of sera (pools of four mice, five pools per group) were determined against four target strains using rabbit sera as the complement source. The table shows geometric mean titres, with the 95% confidence intervals of the mean in parentheses. Mice were given two doses of four dose levels or an adjuvant only control. Note that the data from mice given two doses of 2.5 μg protein per dose is included in Table 4 for comparison, as the immunizations were performed concomitantly.

**Table 3:** Immunogenicity (hSBA) of MenPF-1 vaccine in mice (dose levels 1.0–10.0 μg per dose).

Dose	Serum Bactericidal Titre (GMT) human complement				
μg, (2 doses)	SMenPF1.2 *fetAp*_*17bp*_	H44/76 (WT)	3043 *fetA*::*kan*	3312 *fetAp*_*17bp*,_ *porA*::*ermC*	3311 *fetA*::*kan*, *porA*::*ermC*
1.0	**117** (38–816)	**31** (4–257)	**117** (19–735)	**4** (1–10)	**2** (2–2)
2.5	**407** (140–1181)	**82** (5–1339)	**334** (81–1375)	**3** (1–7)	**2** (2–3)
5.0	**1164** (436–3106)	**110** (12–1023)	**831** (388–1777)	**6** (1–29)	**5** (1–19)
10.0	**1073** (410–2811)	**334** (163–787)	**882** (246–3162)	**4** (1–16)	**3** (2–4)

Serum bactericidal titres of sera (pools of four mice, five pools per group) were determined against four target strains using human sera as the complement source. The table shows geometric mean titres, with the 95% confidence intervals of the mean in parentheses. Mice were given two doses of four dose levels or an adjuvant only control. Note that the data from mice given two doses of 2.5 μg protein per dose is included in Table 5 for comparison, as the immunizations were performed concomitantly.

GMTs for hSBA against strains expressing PorA were significantly higher with 5 or 10 μg doses, than with the 1 μg dose group, P < 0.05 (Table 3). This dose effect, was also observed when rabbit sera was used as a source of complement (P = 0.037), although no significant differences between any pair of doses was found after Tukey’s multiple comparison test was applied.

Against strain 3312 (*porA*::*ermC*), SBA titres were low in all groups, but overall, across all dose levels, rSBA titres were significantly higher than against the corresponding strain 3311 which also lacked expression of FetA (P = 0.007). This difference was not observed with a human source of complement. The highest rSBA titres against 3312 were in sera from mice given 2.5 μg protein per dose (GMT = 25), but overall, in the *porA*::*ermC* there was no significant dose effect (P = 0.071).

The mean SBA titre measured against the *fetA*::*kan*/*porA*::*ermC* strain 3311 was highest for the 10.0 μg dose (rSBA) or 5.0 μg dose (hSBA). Overall, the contribution to bactericidal killing by antigens other than PorA and FetA was negligible.

Mice were also immunized with varying numbers of doses: 1, 2, 3 or 4 doses of 2.5 μg protein per dose. Antibody titres against OMVs, PorA and FetA were low following a single dose (Fig 5B). Although sera from all mice showed measurable responses to the total OMVs, 5/20 mice that received only one dose showed no measurable IgG to either PorA or FetA. Antibody GMTs were similar following 2, 3 or 4 doses. IgG titres against FetA were low regardless of the number of doses administered.

Across all dose numbers, bactericidal activity against strains that did not express PorA (3311 and 3312) was significantly lower in comparison to SMenPF1.2 and 3043. The bactericidal activity of sera against strains expressing PorA (SMenPF1.2 and 3043) was significantly lower following one dose of vaccine compared to multiple doses, rSBA and hSBA: P < 0.05 (Tables 4 and 5). However, the dose effect was more apparent on rSBA titres measured against PorA-negative target strains; following 4 doses, titres were significantly higher than following 1, 2 or 3 doses, rSBA: P < 0.05 (Table 4).

**Table 4:** Immunogenicity (rSBA) of MenPF-1 vaccine in mice (number of doses).

Number of doses	Serum Bactericidal Titre (GMT) rabbit complement			
2.5 μg /dose	SMenPF1.2 *fetAp*_*17bp*_	3043 *fetA*::*kan*	3312 *fetAp*_*17bp*_,*porA*::*ermC*	3311 *fetA*::*kan*, *porA*::*ermC*
1	**253** (200–321)	**32** (19–54)	**11** (9–13)	**6** (5–8)
2	**2097** (1415–3107)	**795** (341–1854)	**25** (14–43)	**7** (5–10)
3	**1683** (996–2844)	**1204** (748–1939)	**22** (15–31)	**11** (7–16)
4	**3366** (2458–4610)	**1552** (950–2535)	**97** (61–155)	**25** (16–38)

Serum bactericidal titres of sera (pools of four mice, five pools per group) were determined against four target strains using rabbit sera as the complement source. The table shows geometric mean titres, with the 95% confidence intervals of the mean in parentheses. Mice were given 1, 2, 3 or 4 doses of 2.5 μg protein per dose. Note that the data from mice given two doses of 2.5 μg protein per dose is included in Table 2 for comparison, as the immunizations were performed concomitantly.

**Table 5:** Immunogenicity (hSBA) of MenPF-1 vaccine in mice (number of doses).

Number of doses	Serum Bactericidal Titre (GMT) human complement				
2.5 μg /dose	SMenPF1.2 *fetAp*_*17bp*_	H44/76 (WT)	3043 *fetA*::*kan*	3312 *fetAp*_*17bp*,_ *porA*::*ermC*	3311 *fetA*::*kan*, *porA*::*ermC*
1	**36** (6–217)	**5** (0–46)	**14** (4–17)	**2** (1–5)	**3** (2–5)
2	**407** (140–1181)	**82** (5–1339)	**334** (81–1375)	**3** (1–7)	**2** (2–3)
3	**507** (235–1094)	**142** (24–857)	**334** (93–1198)	**2** (2–2)	**2** (2–3)
4	**507** (190–1352)	**407** (156–1061)	**221** (107–453)	**2** (1–5)	**2** (2–3)

Serum bactericidal titres of sera (pools of four mice, five pools per group) were determined against four target strains using human sera as the complement source. The table shows geometric mean titres, with the 95% confidence intervals of the mean in parentheses. Mice were given 1, 2, 3 or 4 doses of 2.5 μg protein per dose. Note that the data from mice given two doses of 2.5 μg protein per dose is included in Table 3 for comparison, as the immunizations were performed concomitantly.

Overall for all numbers of doses, in the PorA-negative background rSBA titres were significantly higher when FetA was constitutively expressed (strain 3312) than against 3311 (*fetA*::*kan*), P < 0.001), (Table 4). This strain effect between strains 3312 and 3311 was not replicated when human complement was used in the SBA. For all dose numbers, there was a significant strain effect. Where rabbit complement was used, SBA GMTs were significantly higher against SMenPF1.2 than against the FetA-negative strain 3043; P < 0.05. However after correcting for multiple comparisons using Dunnett’s test, hSBA titres against SMenPF1.2 were not significantly different to those for strain 3043.

Bactericidal titres against the vaccine strain, SMenPF1.2, were compared to the wildtype, unmodified strain H44/76 that expressed a lower level of FetA. Geometric mean hSBA titres were higher against the vaccine strain than unmodified H44/76 for all numbers of doses tested, P < 0.05 (Table 5).

### Immunogenicity in rabbits

**Fig 6:**
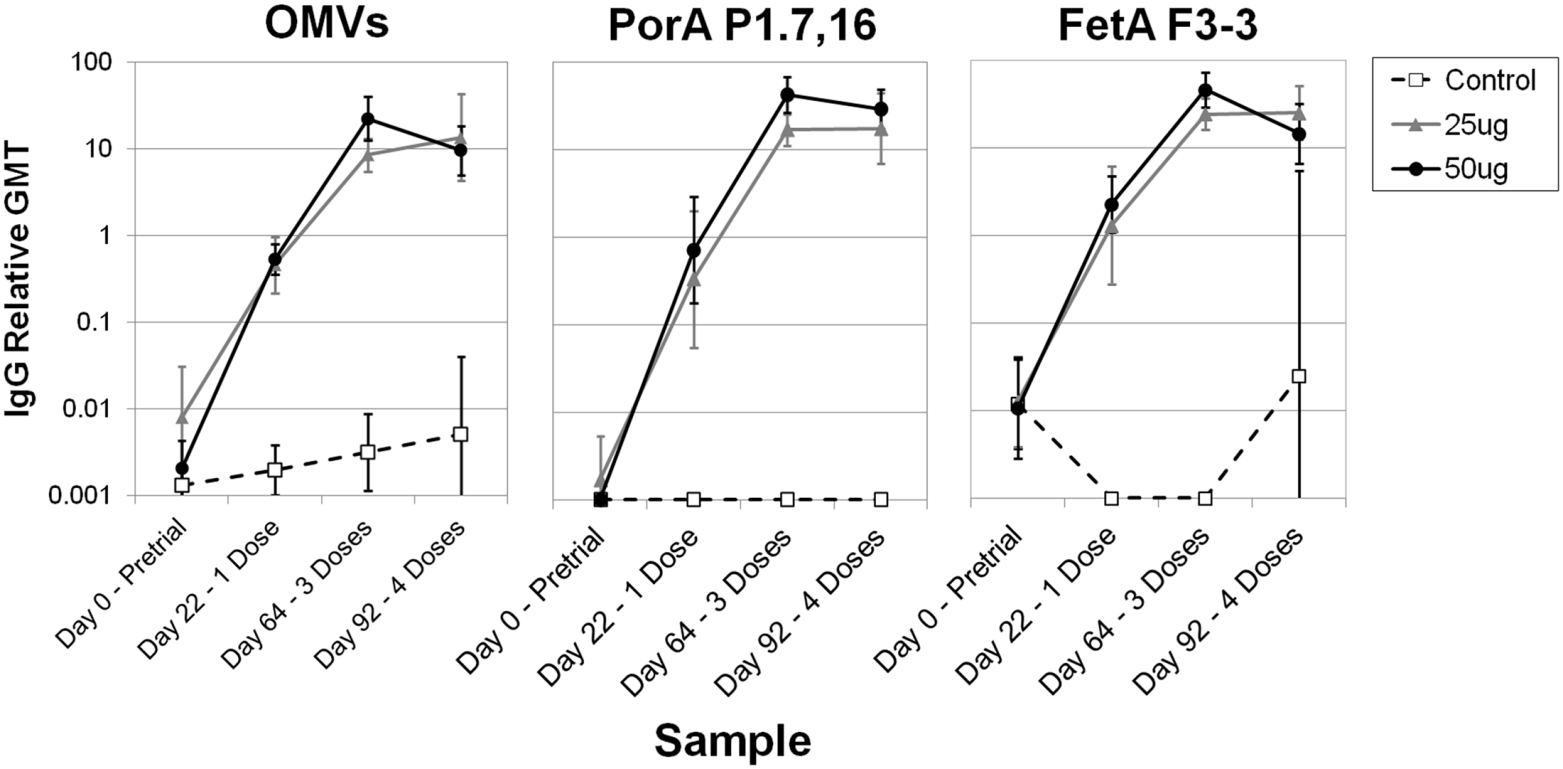
Immunogenicity of strain MenPF-1 in rabbits. Total IgG titres calculated in comparison to a pooled standard serum, were determined for sera from individual rabbits against bulk OMVs and the PorA P1.7,16 and FetA F3-3 antigens. The standard serum was given an arbitrary value of 10 units. Rabbits were immunized with four doses of either adjuvant alone (Control), 25 μg total protein per doses, or 50 μg total protein per doses. Blood samples were collected from all animals (n = 12) pretrial (Sample 1), and before dosing on days 22 (Sample 2, post one dose) and 64 (Sample 3, post three doses). A fourth blood sample was taken from six rabbits on day 92 (Sample 4, post four doses). The graph shows geometric mean units. Error bars indicate the 95% confidence intervals of the mean. Actual figures for the data presented are given in S2 Table.

Sera from the rabbits used in a previous toxicology study (as described in section 2.4 Immunization of rabbits) were assessed for antibody production by ELISA against bulk OMVs, PorA and FetA. Three (main group) or four (recovery group) serum samples were available from each rabbit. The levels of total IgG increased between doses 1 and 3 for all antigens but titres were similar or lower following 4 doses compared to 3 doses (Fig 6). IgG responses were significantly higher in rabbits given 25 μg OMV or 50 μg OMV than those given the adjuvant-only control (Bulk OMV P < 0.001, PorA P < 0.001, FetA P < 0.001, α = 0.025); however, there was no significant difference between the 25 μg and 50 μg treatment groups (OMV P = 0.840, PorA P = 0.262, FetA P = 0.704, α = 0.025).

Immunogenicity in rabbits against both the PorA and FetA antigens was demonstrated satisfactorily.

## Discussion

Assessment of vaccines against highly variable surface antigens using approaches such as serum bactericidal assays (SBAs) requires appropriate controls, ideally highly related isogenic strains that differ only in the vaccine antigens under investigation. Such panels of strains are invaluable, not only in pre-clinical laboratory studies but also in clinical trials in humans. A genetically modified meningococcal strain, which constitutively expressed FetA, was used to produce an OMV for a proof of concept vaccine study along with isogenic strains differing in their ability to express PorA and FetA. The increased expression of FetA resulted in significant enhancement of functional antibody responses, compared to that of the wildtype strain H44/76, indicating that an approach to vaccination using an OMV containing a strong FetA promoter might improve protection. A future development of this concept could be a vaccine containing several additional OMVs each expressing a different PorA and FetA variant to provide comprehensive coverage against hyperinvasive meningococcal serogroup B disease.

PorA is known to be highly immunogenic in outer membrane vesicles, and is immunodominant in all other OMV vaccines. Furthermore, where OMV vaccines have been used against epidemics of meningococcal disease, the protection induced against PorA, as measured by SBA, was later found to have correlated with protection against disease [35]. It has since been agreed by consensus that SBA titres against any protein antigens can be accepted as a correlate of protection for licensure purposes [36]; however, validation of the SBA as a correlate of protection for proteins other than PorA can only be gathered after widespread use of a vaccine based on alternative antigens [37]. This is due to the requirement for a minimum antigen density on the surface of the bacteria for complement-mediated killing. While PorA is expressed at high levels by the majority of meningococcal isolates under most growth conditions, the expression levels of other protein *in vivo* is unknown and often regulated by nutrient availability.

Although available data suggests that FetA is involved in natural immunity generated after invasive disease and during carriage, it is not known whether the level of expression of this antigen *in vivo* is sufficient for complement-mediated killing by specific antibodies. Indeed, the function of FetA remains something of an enigma; unusually for a TonB-coupled iron transporter, it binds iron directly, rather than as part of a siderophore complex [18]. *N*. *meningitidis* is not known to secrete siderophores, and has multiple uptake systems for iron, in the form of transferrin, and lactoferrin-binding proteins, and heme transporters.

Due to the growth of meningococci on iron-replete media, bactericidal antibodies to this protein may not be measured in a typical SBA [38]. FetA-specific antibodies have been shown to be induced following immunization with the New Zealand epidemic OMV vaccine MeNZB (Chiron Vaccines) [39], although these were not shown to be functional. Expression of FetA in MeNZB is also known to have varied among batches [27], so the protection that might have been added by this antigen would have been inconsistent.

In mice, OMVs containing upregulated FetA expressed from a plasmid and lacking PorA have been shown to induce high levels of FetA-specific, bactericidal antibodies [40]. In the present study, in mice, OMVs expressing both PorA and FetA induced high concentrations of antibodies largely directed against PorA. FetA-specific antibodies were also induced, the concentration of which was greatest following four, 2.5 ug doses of vaccine. However, in comparison to the PorA antibodies the concentration of these was low.

The bactericidal activity of the antibodies was determined by SBA against a panel of isogenic strains containing both, one, or none of the two antigens. These showed that the bactericidal killing of the OMV production strain was predominantly dependent on antibodies against PorA and that the antibodies targeting FetA had more limited bactericidal activity. Although the difference in rSBA titres for SMenPF1.2 and 3043 achieved significance across dose numbers, there was no difference in hSBA titres for dose level or dose number data. This is likely due to a lower concentration of antibodies induced against FetA, consistent with the strong immunodominance of PorA. Immunodominance of the response directed to PorA may saturate any differences in bactericidal killing due to levels of FetA expression. This is supported by data obtained in a background of no PorA expression, where the contribution of FetA to bactericidal killing (rSBA) was more apparent with higher levels of bactericidal killing when FetA was expressed (3312) compared to when PorA and FetA were both absent (3311).

There was a positive relationship between the number of doses of vaccine administered and bactericidal activity. This was observable when either human or rabbit complement was used in the assay and in target strains where PorA was present or absent. In addition, increased hSBA titres were associated with higher dose levels in strains expressing PorA. Also noteworthy was killing directed against antigens other than PorA and FetA increased with number of doses, as has been found previously in humans [10].

Given the increased sensitivity of baby rabbit complement to induce bactericidal killing, decreased bactericidal titres were expected when using a human source of complement. The outcome was particularly low hSBA titres for meningococcal strains where PorA was not expressed. However, the analysis was based on a limited number of data points (5 pools of sera per group) and therefore statistical power was limited, particularly after the application of post-hoc tests. Moreover, as mentioned earlier it would be difficult to detect subtle differences resulting from the differing levels of FetA expression when the bactericidal response directed to PorA is so immunodominant. Additionally, there was no effect of dose level on rSBA titres for any of the strains tested. This, together with only seeing a positive effect on titres with greater than 3 doses, would therefore suggest that an optimum dose or schedule may not have been achieved in these particular pre-clinical experiments.

As many other factors are also likely to be important in determining the immunogenicity of the antigens within the OMVs, however, such OMVs should be further tested in humans to compare to these animal models. Although the data show that the vaccine induces FetA-specific bactericidal antibodies, as isogenic mutant strains with constitutively high FetA expression were used for immunological analysis, the conclusions cannot be extrapolated to protection against disease without further evaluation using wildtype meningococci. However, to reliably evaluate the effect of anti-FetA antibodies against wildtype meningococci the classic iron-replete SBA growth conditions would likely have to be modified to enable FetA expression. These modifications would likely affect sensitivity to bactericidal killing in other ways, and as such would also have to be carefully tested.

The induction of protective humoral immunity against the PorA protein using OMVs is widely accepted, and humans are known to generate antibodies against FetA following infection [21]. Here, we have shown that OMVs produced from a meningococcal strain genetically modified to contain constitutive FetA expression induce bactericidal antibodies against both PorA and FetA; however in the case of FetA the bactericidal activity was only detectable when rabbit complement was used. These data indicate that further investigation to determine the potential immunogenicity in humans of a vaccine based on SMenPF1.2 is worthwhile.

## Supporting Information

S1 Fig Plasmid construct pEAT2. (modified from Thompson, 2003). Used in the construction of meningococcal strain 3043. The *fetA* gene was cloned into plasmid vector pTrcHis2 (Invitrogen). A kanamycin resistance cassette amplified by PCR from the Tn5 transposon was introduced into a unique *Eco*O109I restriction site.
(DOCX)

S2 Fig Structure of plasmid PorA-EryF. Used for transformation of meningococcal strains SMenPF1.2 and 3043 to generate PorA mutations in strains 3311 and 3312 respectively. The porA gene was interrupted at a *Bsi*WI site by insertion of an erythromycin-resistance cassette sub-cloned from plasmid pER2 (van der Voort et al., 1986).
(DOCX)

S1 Table Immunogenicity of MenPF-1 in mice. Total IgG titres, calculated in comparison to a pooled standard serum, were determined for sera from individual mice against bulk OMVs and the PorA and FetA antigens. The standard serum was given an arbitrary value of 10 units. The table shows the geometric mean IgG titres, with the 95% confidence intervals of the mean shown below in parentheses.
(DOCX)

S2 Table Immunogenicity of MenPF-1 in rabbits. Total IgG titres, calculated in comparison to a pooled standard serum were determined for sera from individual rabbits against bulk OMVs and the PorA and FetA antigens. The standard serum was given an arbitrary value of 10 units. The table shows the geometric mean IgG titres, with the 95% confidence intervals of the mean shown below in parentheses.
(DOCX)

## References

[1] ECDC (2013) Annual Epidemiological Report 2012: Reporting on 2010 surveillance data and 2011 epidemic intelligence data. Stockholm: ECDC.

[2] Yongye AB, Gonzalez-Outeirino J, Glushka J, Schultheis V, Woods RJ (2008) The conformational properties of methyl alpha-(2,8)-di/trisialosides and their N-acyl analogues: implications for anti-Neisseria meningitidis B vaccine design. Biochemistry 47: 12493–12514. 10.1021/bi800431c 18954144 PMC2957299

[3] Finne J, Leinonen M, Makela PH (1983) Antigenic similarities between brain components and bacteria causing meningitis. Implications for vaccine development and pathogenesis. Lancet 2: 355–357. 6135869 10.1016/s0140-6736(83)90340-9

[4] Holst J (2007) Strategies for development of universal vaccines against meningococcal serogroup B disease: the most promising options and the challenges evaluating them. Hum Vaccin 3: 290–294. 17712231 10.4161/hv.4513

[5] Sierra GV, Campa HC, Varcacel NM, Garcia IL, Izquierdo PL, Sotolongo PF, et al (1991) Vaccine against group B Neisseria meningitidis: protection trial and mass vaccination results in Cuba. NIPH Ann 14: 195–207; discussion 208–110. 1812432

[6] de Moraes JC, Perkins BA, Camargo MC, Hidalgo NT, Barbosa HA, Sacchi CT, et al (1992) Protective efficacy of a serogroup B meningococcal vaccine in Sao Paulo, Brazil. Lancet 340: 1074–1078. 1357461 10.1016/0140-6736(92)93086-3

[7] Arnold R, Galloway Y, McNicholas A, O'Hallahan J (2011) Effectiveness of a vaccination programme for an epidemic of meningococcal B in New Zealand. Vaccine 29: 7100–7106. 10.1016/j.vaccine.2011.06.120 21803101

[8] Bjune G, Hoiby EA, Gronnesby JK, Arnesen O, Fredriksen JH, Halstensen A, et al (1991) Effect of outer membrane vesicle vaccine against group B meningococcal disease in Norway. Lancet 338: 1093–1096. 1682541 10.1016/0140-6736(91)91961-s

[9] Caron F, Delbos V, Houivet E, Deghmane AE, Leroy JP, Hong E, et al (2012) Evolution of immune response against Neisseria meningitidis B:14:P1.7,16 before and after the outer membrane vesicle vaccine MenBvac. Vaccine 30: 5059–5062. 10.1016/j.vaccine.2012.05.051 22658929

[10] Rosenqvist E, Hoiby EA, Wedege E, Bryn K, Kolberg J, Klem A, et al (1995) Human antibody responses to meningococcal outer membrane antigens after three doses of the Norwegian group B meningococcal vaccine. Infect Immun 63: 4642–4652. 7591118 10.1128/iai.63.12.4642-4652.1995 PMC173667

[11] Feavers IM, Pizza M (2009) Meningococcal protein antigens and vaccines. Vaccine 27 Suppl 2: B42–50. 10.1016/j.vaccine.2009.05.001 19481315

[12] Serruto D, Bottomley MJ, Ram S, Giuliani MM, Rappuoli R (2012) The new multicomponent vaccine against meningococcal serogroup B, 4CMenB: immunological, functional and structural characterization of the antigens. Vaccine 30 Suppl 2: B87–97. 10.1016/j.vaccine.2012.01.033 22607904 PMC3360877

[13] van den Dobbelsteen GP, van Dijken HH, Pillai S, van Alphen L (2007) Immunogenicity of a combination vaccine containing pneumococcal conjugates and meningococcal PorA OMVs. Vaccine 25: 2491–2496. 17023098 10.1016/j.vaccine.2006.09.025

[14] Cartwright K, Morris R, Rumke H, Fox A, Borrow R, Begg N, et al (1999) Immunogenicity and reactogenicity in UK infants of a novel meningococcal vesicle vaccine containing multiple class 1 (PorA) outer membrane proteins. Vaccine 17: 2612–2619. 10418910 10.1016/s0264-410x(99)00044-4

[15] Weynants VE, Feron CM, Goraj KK, Bos MP, Denoel PA, Verlant VG, et al (2007) Additive and synergistic bactericidal activity of antibodies directed against minor outer membrane proteins of Neisseria meningitidis. Infect Immun 75: 5434–5442. 17664268 10.1128/IAI.00411-07 PMC2168297

[16] Welsch JA, Ram S, Koeberling O, Granoff DM (2008) Complement-dependent synergistic bactericidal activity of antibodies against factor H-binding protein, a sparsely distributed meningococcal vaccine antigen. J Infect Dis 197: 1053–1061. 10.1086/528994 18419542

[17] Comanducci M, Bambini S, Caugant DA, Mora M, Brunelli B, Capecchi B, et al (2004) NadA diversity and carriage in Neisseria meningitidis. Infect Immun 72: 4217–4223. 15213166 10.1128/IAI.72.7.4217-4223.2004 PMC427459

[18] Saleem M, Prince SM, Rigby SE, Imran M, Patel H, Chan H, et al (2013) Use of a molecular decoy to segregate transport from antigenicity in the FrpB iron transporter from Neisseria meningitidis. PLoS One 8: e56746 10.1371/journal.pone.0056746 23457610 PMC3574120

[19] Tanabe M, Nimigean CM, Iverson TM (2010) Structural basis for solute transport, nucleotide regulation, and immunological recognition of Neisseria meningitidis PorB. Proc Natl Acad Sci U S A 107: 6811–6816. 10.1073/pnas.0912115107 20351243 PMC2872391

[20] Wedege E, Bolstad K, Wetzler LM, Guttormsen H (2000) IgG antibody levels to meningococcal porins in patient sera: comparison of immunoblotting and ELISA measurements. J Immunol Methods 244: 9–15. 11033014 10.1016/s0022-1759(00)00245-3

[21] Ala'Aldeen DA, Wall RA, Borriello SP (1990) Immunogenicity and cross-reactivity of the 70-Kda iron-regulated protein of Neisseria meningitidis in man and animals. J Med Microbiol 32: 275–281. 2118187 10.1099/00222615-32-4-275

[22] Brehony C, Jolley KA, Maiden MC (2007) Multilocus sequence typing for global surveillance of meningococcal disease. FEMS Microbiol Rev 31: 15–26. 17168997 10.1111/j.1574-6976.2006.00056.x

[23] Buckee CO, Jolley KA, Recker M, Penman B, Kriz P, Gupta S, et al (2008) Role of selection in the emergence of lineages and the evolution of virulence in Neisseria meningitidis. Proc Natl Acad Sci U S A 105: 15082–15087. 10.1073/pnas.0712019105 18815379 PMC2553036

[24] Watkins ER, Maiden MC (2012) Persistence of hyperinvasive meningococcal strain types during global spread as recorded in the PubMLST database. PLoS One 7: e45349 10.1371/journal.pone.0045349 23028953 PMC3460945

[25] Urwin R, Russell JE, Thompson EA, Holmes EC, Feavers IM, Maiden MC (2004) Distribution of surface protein variants among hyperinvasive meningococci: implications for vaccine design. Infect Immun 72: 5955–5962. 15385499 10.1128/IAI.72.10.5955-5962.2004 PMC517544

[26] Ala'Aldeen DA, Davies HA, Borriello SP (1994) Vaccine potential of meningococcal FrpB: studies on surface exposure and functional attributes of common epitopes. Vaccine 12: 535–541. 7518627 10.1016/0264-410x(94)90314-x

[27] Vipond C, Wheeler JX, Jones C, Feavers IM, Suker J (2005) Characterization of the protein content of a meningococcal outer membrane vesicle vaccine by polyacrylamide gel electrophoresis and mass spectrometry. Hum Vaccin 1: 80–84. 17038831 10.4161/hv.1.2.1651

[28] Thompson EA (2001) Antigenic variation in the potential meningococcal vaccine candidate FetA. Oxford: PhD Thesis, University of Oxford.

[29] van der Voort ER, van der Ley P, van der Biezen J, George S, Tunnela O, van Dijken H, et al (1996) Specificity of human bactericidal antibodies against PorA P1.7,16 induced with a hexavalent meningococcal outer membrane vesicle vaccine. Infect Immun 64: 2745–2751. 8698504 10.1128/iai.64.7.2745-2751.1996 PMC174135

[30] Frasch CE, van Alphen L, Holst J, Poolman JT, Rosenqvist E (2001) Outer membrane protein vesicle vaccines for meningococcal disease. Methods Mol Med 66: 81–107. 10.1385/1-59259-148-5:81 21336749

[31] Norheim G, Tunheim G, Naess LM, Kristiansen PA, Caugant DA, Rosenqvist E (2012) An outer membrane vesicle vaccine for prevention of serogroup A and W-135 meningococcal disease in the African meningitis belt. Scand J Immunol 76: 99–107. 10.1111/j.1365-3083.2012.02709.x 22537024

[32] Saleem M, Prince SM, Patel H, Chan H, Feavers IM, Derrick JP (2012) Refolding, purification and crystallization of the FrpB outer membrane iron transporter from Neisseria meningitidis. Acta Crystallogr Sect F Struct Biol Cryst Commun 68: 231–235. 10.1107/S1744309111056028 22298007 PMC3274411

[33] Saleem M, Moore J, Derrick JP (2012) Expression, purification and crystallization of Neisserial outer membrane proteins In: Christodoulides M, editor. Neisseria meningitidis: Advanced Methods and Protocols. New York: Springer pp. 91–106. 10.1007/978-1-61779-346-2_6 21993641

[34] Borrow R, Aaberge IS, Santos GF, Eudey TL, Oster P, Glennie A, et al (2005) Interlaboratory standardization of the measurement of serum bactericidal activity by using human complement against meningococcal serogroup b, strain 44/76-SL, before and after vaccination with the Norwegian MenBvac outer membrane vesicle vaccine. Clin Diagn Lab Immunol 12: 970–976. 16085915 10.1128/CDLI.12.8.970-976.2005 PMC1182195

[35] Holst J, Feiring B, Fuglesang JE, Hoiby EA, Nokleby H, Aaberge IS, et al (2003) Serum bactericidal activity correlates with the vaccine efficacy of outer membrane vesicle vaccines against Neisseria meningitidis serogroup B disease. Vaccine 21: 734–737. 12531351 10.1016/s0264-410x(02)00591-1

[36] Frasch CE, Borrow R, Donnelly J (2009) Bactericidal antibody is the immunologic surrogate of protection against meningococcal disease. Vaccine 27 Suppl 2: B112–116. 10.1016/j.vaccine.2009.04.065 19464093

[37] Vipond C, Care R, Feavers IM (2012) History of meningococcal vaccines and their serological correlates of protection. Vaccine 30 Suppl 2: B10–17. 10.1016/j.vaccine.2011.12.060 22607894

[38] Wedege E, Hoiby EA, Rosenqvist E, Bjune G (1998) Immune responses against major outer membrane antigens of Neisseria meningitidis in vaccinees and controls who contracted meningococcal disease during the Norwegian serogroup B protection trial. Infect Immun 66: 3223–3231. 9632589 10.1128/iai.66.7.3223-3231.1998 PMC108336

[39] Wedege E, Bolstad K, Aase A, Herstad TK, McCallum L, Rosenqvist E, et al (2007) Functional and specific antibody responses in adult volunteers in new zealand who were given one of two different meningococcal serogroup B outer membrane vesicle vaccines. Clin Vaccine Immunol 14: 830–838. 17494638 10.1128/CVI.00039-07 PMC1951067

[40] Kortekaas J, Pettersson A, van der Biezen J, Weynants VE, van der Ley P, Poolman J, et al (2007) Shielding of immunogenic domains in Neisseria meningitidis FrpB (FetA) by the major variable region. Vaccine 25: 72–84. 16914236 10.1016/j.vaccine.2006.07.016

