# Supplementary material for: FetA Antibodies Induced by an Outer Membrane Vesicle Vaccine Derived from a Serogroup B Meningococcal Isolate with Constitutive FetA Expression
Source: PLoS One. 2015 Oct 14;10(10):e0140345. doi: 10.1371/journal.pone.0140345 (PMC4605655; doi:10.1371/journal.pone.0140345)
Supplement: S1 Fig — (modified from Thompson, 2003). Used in the construction of meningococcal strain 3043. The fetA gene was cloned into plasmid vector pTrcHis2 (Invitrogen). A kanamycin resistance cassette amplified by PCR from the Tn5 transposon was introduced into a unique EcoO109I restriction site. (DOCX) [file pone.0140345.s001.docx]

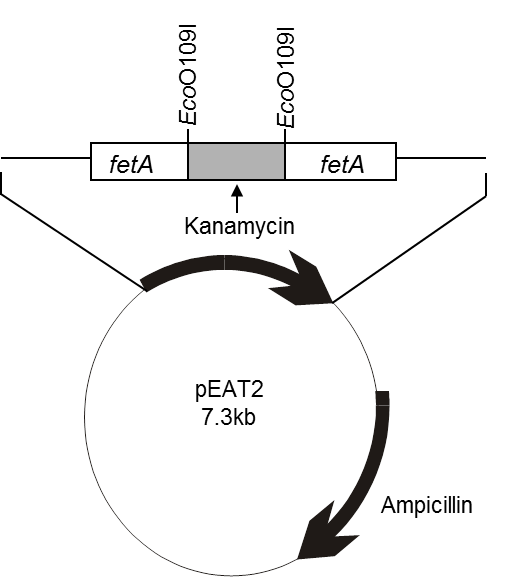


**S1 Fig. Plasmid construct pEAT2.** (modified from Thompson, 2003). Used in the construction of meningococcal strain 3043. The *fetA* gene was cloned into plasmid vector pTrcHis2 (Invitrogen). A kanamycin resistance cassette amplified by PCR from the Tn5 transposon was introduced into a unique *Eco*O109I restriction site.
