# Supplementary material for: FetA Antibodies Induced by an Outer Membrane Vesicle Vaccine Derived from a Serogroup B Meningococcal Isolate with Constitutive FetA Expression
Source: PLoS One. 2015 Oct 14;10(10):e0140345. doi: 10.1371/journal.pone.0140345 (PMC4605655; doi:10.1371/journal.pone.0140345)
Supplement: S2 Fig — Used for transformation of meningococcal strains SMenPF1.2 and 3043 to generate PorA mutations in strains 3311 and 3312 respectively. The porA gene was interrupted at a BsiWI site by insertion of an erythromycin-resistance cassette sub-cloned from plasmid pER2 (van der Voort et al., 1986). (DOCX) [file pone.0140345.s002.docx]

**S2 Fig. Structure of plasmid PorA-EryF.** Used for transformation of meningococcal strains SMenPF1.2 and 3043 to generate PorA mutations in strains 3311 and 3312 respectively. The *porA* gene was interrupted at a *Bsi*WI site by insertion of an erythromycin-resistance cassette sub-cloned from plasmid pER2 [29].
