# Supplementary material for: FetA Antibodies Induced by an Outer Membrane Vesicle Vaccine Derived from a Serogroup B Meningococcal Isolate with Constitutive FetA Expression
Source: PLoS One. 2015 Oct 14;10(10):e0140345. doi: 10.1371/journal.pone.0140345 (PMC4605655; doi:10.1371/journal.pone.0140345)
Supplement: S2 Table — Total IgG titres, calculated in comparison to a pooled standard serum were determined for sera from individual rabbits against bulk OMVs and the PorA and FetA antigens. The standard serum was given an arbitrary value of 10 units. The table shows the geometric mean IgG titres, with the 95% confidence intervals of the mean shown below in parentheses. (DOCX) [file pone.0140345.s004.docx]

**S2 Table. Immunogenicity of MenPF-1 in rabbits**.

| **Group** | **Sample** | **Relative IgG** | | |
| --- | --- | --- | --- | --- |
|  |  | **Total OMV** | **PorA P1.7,16** | **FetA F3-3** |
| **Control** | **Day 0** | 0.001  (0.001 – 0.002) | 0.001  (0.001 – 0.001) | 0.012  (0.004 – 0.039) |
|  | **Day 22** | 0.002  (0.001 - 0.004) | 0.001  (0.001 – 0.001) | 0.001  (0.001 – 0.001) |
|  | **Day 64** | 0.003  (0.001 – 0.009) | 0.001  (0.001 – 0.001) | 0.001  (0.001 – 0.001) |
|  | **Day 92** | 0.005  (0.001 – 0.040) | 0.001  (0.001 – 0.001) | 0.025  (0.000 – 5.509) |
| **25 µg** | **Day 0** | 0.008  (0.002 – 0.031) | 0.002  (0.001 – 0.005) | 0.013  (0.004 – 0.042) |
|  | **Day 22** | 0.459  (0.217 – 0.092) | 0.331  (0.055 – 2.002) | 1.302  (0.276 – 6.135) |
|  | **Day 64** | 8.483  (5.463 – 13.172) | 16.818  (11.078 – 25.530) | 24.493  (16.211 – 37.006) |
|  | **Day 92** | 13.486  (4.283 – 42.471) | 17.431  (6.880 – 44.165) | 25.226  (12.495 – 50.926) |
| **50 µg** | **Day 0** | 0.002  (0.001 – 0.004) | 0.001  (0.001 – 0.001) | 0.011  (0.003 – 0.040) |
|  | **Day 22** | 0.530  (0.353 – 0.795) | 0.715  (0.177 – 2.887) | 2.293  (1.098 – 4.790) |
|  | **Day 64** | 22.184  (12.466 – 39.478) | 42.627  (26.716 – 68.015) | 46.343  (29.378 – 73.102) |
|  | **Day 92** | 9.574  (4.975 – 18.422) | 29.287  (17.416 – 49.250) | 14.663  (6.718 – 32.008) |

Total IgG titres, calculated in comparison to a pooled standard serum, were determined for sera from individual rabbits against bulk OMVs and the PorA and FetA antigens. The standard serum was given an arbitrary value of 10 units. The table shows the geometric mean IgG titres, with the 95% confidence intervals of the mean shown below in parentheses.
